# Morphology and Updated Phylogeny Revealed Three New Species in *Chlorencoelia* (Cenangiaceae, Helotiales) from China

**DOI:** 10.3390/jof11120855

**Published:** 2025-12-01

**Authors:** Huan-Di Zheng, Wen-Ying Zhuang

**Affiliations:** State Key Laboratory of Microbial Diversity and Innovative Utilization, Institute of Microbiology, Chinese Academy of Sciences, Beijing 100101, China; zhuangwy@im.ac.cn

**Keywords:** geographical distribution, inoperculate cup-fungi, Leotiomycetes, morphology, phylogeny

## Abstract

Morphological and molecular phylogenetic studies of *Chlorencoelia* in China revealed three novel species, which are described and illustrated as *C. biguttulata*, *C. ellipsoidea*, and *C. sinensis*. *Chlorencoelia biguttulata* is characterized by greyish green to blackish green hymenium surface, clavate tomentum hyphae on receptacle surface, and inequilateral ellipsoidal ascospores having two large guttules and 8.5–14.5 × 3.5–4.5 μm. *Chlorencoelia ellipsoidea* differs from the known species by distinctly stipitate apothecia with a dark green hymenium surface and ellipsoidal ascospores 7.2–9.1 × 3.5–4.2 μm. *Chlorencoelia sinensis* is characterized by substipitate apothecia, blackish receptacle surface, and fusoidal ascospores 9–13.7 × 2.2–3.5 μm. Sequence analyses of the ITS region supported recognition of them as separate species. All three new species occurred on rotten wood in montane forests. Morphological features for species delimitation and diversity of *Chlorencoelia* are discussed. An identification key to the known species of the genus is provided.

## 1. Introduction

The genus *Chlorencoelia* J.R. Dixon was introduced by Dixon in 1975, and typified by *C. versiformis* (Pers.) J.R. Dixon, which included *C. torta* (Schwein.) J.R. Dixon as well [1]. The two species were once treated as members of *Chlorosplenium* Fr. (Chlorospleniaceae) and *Chlorociboria* Seaver ex C.S. Ramamurthi, Korf & L.R. Batra (Chlorociboriaceae). Thereafter, *C. indica* (K.S. Thind, E.K. Cash & Pr. Singh) W.Y. Zhuang was transferred into the genus [2]. Subsequently, *C. ripakorfii* Iturr. & Mardones from Venezuela and *C. macrospora* F. Ren & W.Y. Zhuang from China were added [3,4]. New members were not reported in the following 10 years (2015–2024). Recently, Patejuk et al. introduced two new species from New Zealand, *C. australis* P.R. Johnst. & Patejuk and *C. northlandica* P.R. Johnst. & Patejuk, as well as a new combination, *C. olivacea* (Rodway) P.R. Johnst. & Patejuk., on the basis of integrative study of DNA sequences and morphology [5]. Currently, the genus comprises eight known species.

*Chlorencoelia* was originally regarded as a member of the family Helotiaceae [1]. Later, it was placed in the family Hemiphacidiaceae based on phylogenetic analyses of rDNA sequences [6,7,8]. The multi-locus phylogeny revealed that members of Hemiphacidiaceae and the core group of the subfamily Encoelioideae (including *Chlorencoelia*) belonged to the resurrected family Cenangiaceae [9], which was confirmed by the subsequent studies [10,11,12,13,14].

*Chlorencoelia* is characterized by shallow cupulate to infundibuliform apothecia, mostly less than 10 mm in diam., sub-stipitate to stipitate, solitary to gregarious; hymenium surface olive-yellow, olive-green to dark green; receptacle surface dark olive-green to blackish; ectal excipulum of hyaline- to brown-celled textura angularis, giving rise to clavate or filamentous tomentum hyphae; medullary excipulum of textura intricata with cells having hyaline to dark brown walls; asci cylindric-clavate, J+, 8-spored; ascospores ellipsoid, cylindric-oblong to allantoid, hyaline, guttulate; paraphyses filiform, septate, with pale yellow to green vacuolar bodies. Shape and size of ascospores, asci, and tomentum hyphae are key characteristics for species delimitation [1,3,4,5].

*Chlorencoelia* species are saprotrophic on rotten wood of angiosperm and gymnosperm trees. *C. versiformis* was collected on *Betula*, *Nothofagus*, *Quercus*, *Tsuga*, and unidentified wood [1]. *C. torta* was found on *Acer*, *Betula*, *Fagus*, *Quercus*, *Tabebuia*, coniferous, and unidentified wood [1]. *C. indica* was recorded on *Cedrus deodara* [2]. *C. australis* grew on *Fuscospora solandri*, *Leptospermum scoparium*, *Nothofagus*, Nothofagaceae, and unidentified wood [5]. *C. olivacea* occurred on *Fuscospora fusca*, *Fuscospora cliffortioides*, *Nothofagus*, *Pterophylla racemosa*, Nothofagaceae, and unidentified wood [5]. *C. macrospora*, *C. northlandica*, and *C. ripakorfii* on woody substrate were not clearly documented. The correlations between *Chlorencoelia* species and their tree substrates were not confirmed.

Three *Chlorencoelia* species, *C. macrospora*, *C. torta*, and *C. versiformis*, were previously reported from China [4,15,16]. During the diversity surveys of helotialean fungi in recent years, additional specimens of *Chlorencoelia* were collected. The identities of those collections were determined based on morphological features and DNA sequences. It turns out that three new species were discovered. The previous records of *C. torta* and *C. versiformis* in China were based on misidentifications, and should be excluded from the Chinese mycobiota.

## 2. Materials and Methods

### 2.1. Morphological Observations

Specimens of *Chlorencoelia* preserved in the Herbarium Mycologicum Academiae Sinicae (HMAS) were loaned and re-examined. New specimens were collected on decorticated rotten wood from Chongqing City, Xizang, and Yunnan during 2016–2020. Due to the excessive decayed substrate, it is difficult to recognize the tree species. The substrates were simply recorded as decorticated rotten wood. Fresh apothecia were dried at about 50 °C. Macroscopic photographs were taken with a Canon G16 digital camera (Canon Inc., Tokyo, Japan). Macroscopic characteristics were recorded according to the field notes or photographs of fresh apothecia. Dry specimens were observed under a SZX7 stereomicroscope (Olympus Corporation, Tokyo, Japan). Dried apothecia were soaked in distilled water for rehydration, and longitudinal sections were made using a Yidi YD-1508A freezing microtome (Jinhua Yidi Medical Appliance Co., Ltd., Jinhua, China) at a thickness of 15−20 μm. Amyloid reactions of ascus apical rings were examined in Lugol’s solution and Melzer’s reagent with or without pretreatment of 3% KOH solution. Microscopic examinations and measurements were carried out from sections and squash mounts in lactophenol cotton blue solution via an Olympus BH-2 microscope (Olympus Corporation, Tokyo, Japan). A ZEISS Axiocam 305 colour microscope camera (Carl Zeiss AG, Göttingen, Germany) attached to a Zeiss Axioskop 2 Plus microscope (Göttingen, Germany) was used to take microscopic photographs. Newly collected specimens were deposited in HMAS.

### 2.2. DNA Extraction, Amplification, and Sequencing

Plant Genomic DNA Kit (TIANGEN Biotech. Co., Beijing, China) was used to extract genomic DNA from dried apothecia following the manufacturer’s instructions. The primer pairs used for amplification and sequencing are ITS1&ITS4 or ITS5&ITS4 for internal transcribed spacers region of the ribosomal DNA (ITS) [17], LR0R/LR5 for the D1/D2 region of large subunit ribosomal DNA (28S) [18,19], RPB1-Af/RPB1-Cr for RNA polymerase II largest subunit gene (RPB1) [20,21], fRPB2-5f/fRPB2-7cr for RNA polymerase II second largest subunit gene (RPB2) [22], and EF1-983F/EF1-1567R for translation elongation factor 1-alpha gene (TEF1) [23]. Applied Biosystems 2720 thermocycler (Foster City, CA, USA) was used for PCR reactions following the components and cycling parameters adopted by Zheng & Zhuang 2022 [24]. The PCR products were purified and sequenced by Beijing Tianyi Huiyuan Bioscience and Technology, Beijing, China.

### 2.3. Sequence Assembly, Alignment and Phylogenetic Analyses

Forward and reverse sequences generated were assembled with BioEdit 7.0.5.3 [25]. Newly generated sequences were submitted to GenBank and provided as Supplementary Files (S1–S5) as some sequence accession numbers are still pending. Other sequences used for phylogenetic analyses were downloaded from GenBank. *Hymenoscyphus fraxineus* (T. Kowalski) Baral, Queloz & Hosoya and *H. fructigenus* (Bull.) Gray were chosen as the outgroup taxa. The accession numbers and related information of the ITS sequences used in phylogenetic analyses are presented in Table 1. Sequences were aligned and optimized through BioEdit 7.0.5.3 [25]. GenBank accession numbers of the newly generated RPB1, RPB2, and TEF1 sequences are provided following the voucher specimen numbers for future phylogenetic consideration.

Bayesian inference (BI) analysis was performed with MrBayes 3.1.2 [26], and Maximum likelihood (ML) analysis was conducted using RAxML 8.0 [27]. The best-fit substitution model for the ITS matrix was calculated based on the Akaike Information Criterion (AIC) with MrModeltest 2.3 [28]. For BI analysis, two parallel runs were conducted. Each run contained four simultaneous Markov Chain Monte Carlo (MCMC) chains. MCMC chains were run for 2 million generations with trees sampled at 100-generation intervals. The first 25% of the trees were discarded as burn-in, and the remaining trees were used to calculate the posterior probabilities (PP) of the majority rule consensus tree. ML analysis was run in RAxML 8.0 using a GTRGAMMA model and tested with 1000 nonparametric bootstrap replicates [27]. The phylogenetic trees were visualized in FigTree 1.4.4 [29].

## 3. Results

### 3.1. Phylogenetic Analyses

All the ITS sequences labelled as *Chlorencoelia* in the GenBank database were downloaded and evaluated. Except for some wrongly identified ones [e.g., an endophytic fungal strain (CMON55, ITS JQ754001) of common bean (*Phaseolus vulgaris*) leaves and two endophytic strains (FeC10, ITS MW447089 and FeC18, ITS MW447090)] and those shorter than 300 bp, others joined the phylogenetic analyses together with the newly generated ones.

**Figure 1:**
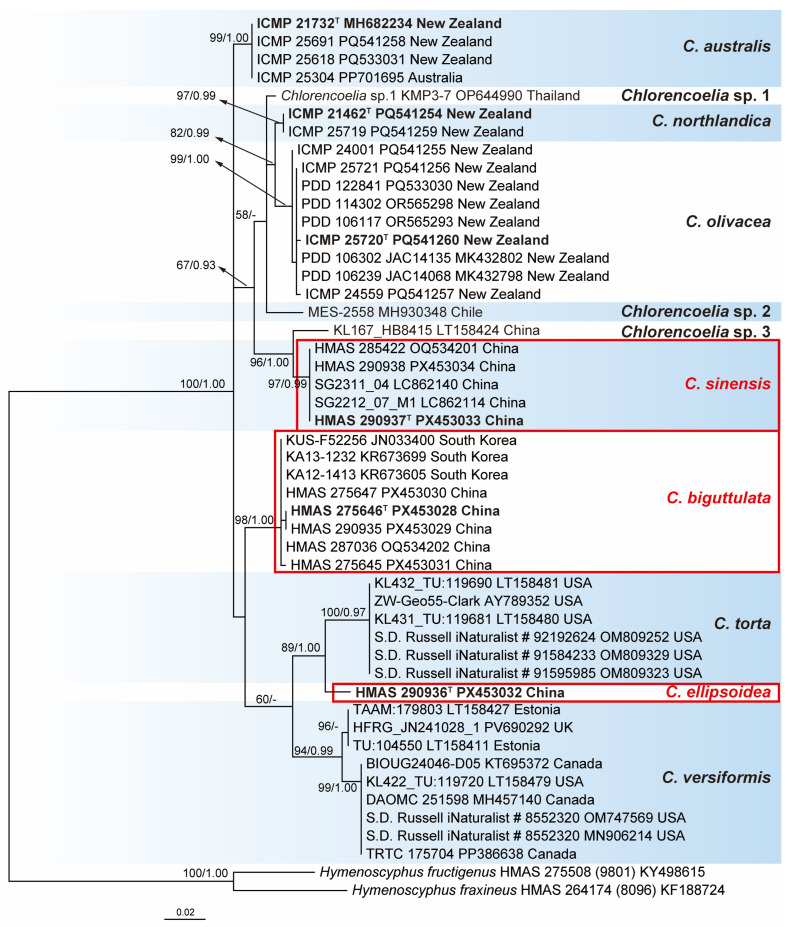
Maximum likelihood phylogenetic tree of *Chlorencoelia* inferred from ITS sequences. Bootstrap support values ≥ 50% (left) of ML and posterior probability values ≥ 0.90 (right) of BI analysis are shown at nodes. Holotype specimens or ex-type strains are shown in bold and marked with superscript letter “T”. New species are encircled by red boxes. The origins of the materials are indicated at the end of each sample.

The ITS dataset consisted of 49 sequences representing 11 taxa of *Chlorencoelia* with two *Hymenoscyphus* species as outgroup taxa. The alignment consisted of 526 characters, eliminating poorly aligned regions, including 373 constant ones, 52 variable ones, and 101 parsimony-informative ones. SYM + G was selected as the best-fit model for BI analysis. The topologies of the ML and BI trees are similar, and only the phylogram of the ML tree is shown in Figure 1.

All the sampled sequences of *Chlorencoelia* formed a well-supported monophyletic group (100% MLBP and 1.00 BIPP). Eleven species-level clades are recognized within the genus, which correspond to the eight known species (including the proposed three new species) and three undescribed taxa. The type locality of *C. torta* is the USA [1]. Sequences of *C. torta* derived from North American materials were aggregated together to form a separate clade and thus considered as reliable representatives of the species. The type locality of *C. versiformis* is uncertain and was presumed to be Germany [1], and sequences under that name derived from European (Estonian and UK) materials were represented as an independent species. As for *C. versiformis*, two subclades were recognized, one comprising the European samples and the other from North America. Sequences of *C. australis*, *C. northlandica*, and *C. olivacea* from New Zealand, as well as those of the to-be-described new species, clustered as six well-supported clades and recognized at species rank. The remaining three terminal lineages, “*Chlorencoelia* sp.1 KMP3-7 OP644990” (as *C. torta* in GenBank) from Thailand, “*Chlorencoelia* sp.2 MES-2558 MH930348” from Chile (as cf. *Chlorencoelia* sp. in GenBank), and “*Chlorencoelia* sp.3 KL167_HB8415 LT158424” (as *C. torta* in GenBank) from China, each having a single sequence, are presumably putative new species in view of the tree topology (Figure 1).

### 3.2. Taxonomy

#### 3.2.1. *Chlorencoelia biguttulata* H.D. Zheng & W.Y. Zhuang, sp. nov. (Figure 2a–c and Figure 3)

Fungal Names—FN 573049.

Etymology—The specific epithet refers to the two large guttules in ascospores of the fungus.

Holotype—China, Xizang, Nyingchi, Lulang, on decorticated rotten wood, 23 September 2016, Huan-Di Zheng, Zhi-He Yu, Zhao-Qing Zeng et al. 11236 (HMAS 275646; ITS: PX453028, 28S: PX630171, RPB1: PX557843, RPB2: PX557850, TEF1: PX528999).

Chinese name—双油滴绿散孢盘菌, shuāng yóu dī lǜ sǎn bāo pán jūn.

**Figure 2:**
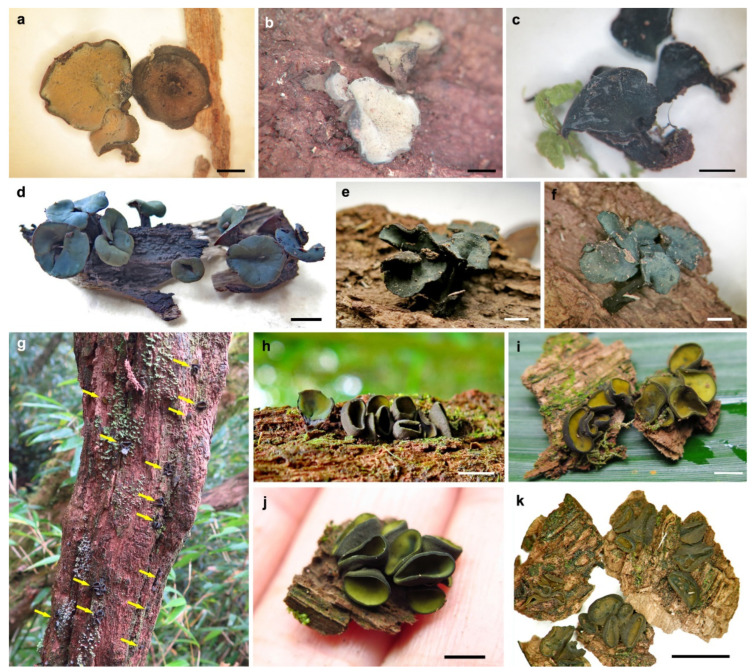
Apothecia of *Chlorencoelia* spp. on woody substrates ((**a**–**c**,**e**,**f**,**k**). dry apothecia; (**d**,**g**–**j**). fresh apothecia). (**a**–**c**) *C. biguttulata*, (**a**,**b**) HMAS 275646, holotype; (**c**) HMAS 275645, paratype; (**d–f**) *C. ellipsoidea*, HMAS 290936, holotype; (**g**–**k**) *C. sinensis*, HMAS 290937, holotype. Scale bars: (**a**–**c**,**e**,**f**,**h**–**j**) = 2 mm; (**d**,**k**) = 5 mm.

**Figure 3:**
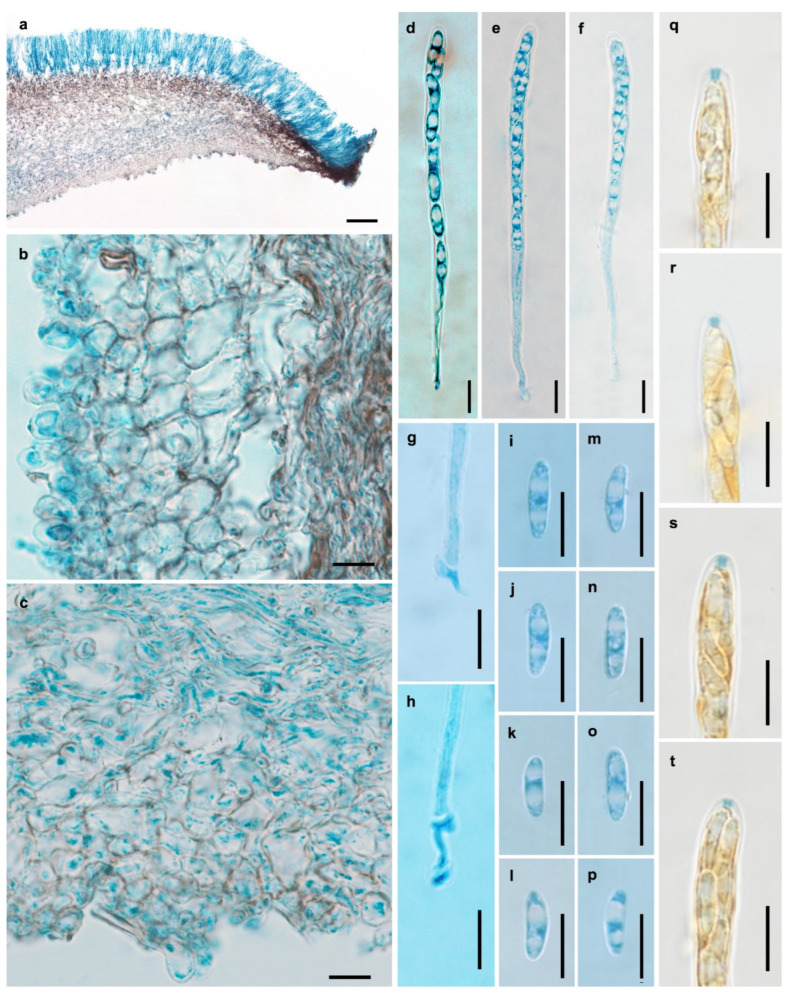
Microscopic features of *Chlorencoelia biguttulata* (HMAS 275646, holotype). (**a**) Longitudinal section of apothecium (partial); (**b**,**c**) excipular structure of flank; (**d**–**f**) asci; (**g**,**h**) crozier at ascus base; (**i**–**p**) ascospores; (**q**–**t**) IKI reaction of ascus apical ring without KOH pretreatment. Scale bars: (**a**) = 100 μm; (**b**–**t**) = 10 μm. Mounting media: (**a**–**p**) lactophenol cotton blue; (**q**–**t**) Melzer’s reagent.

Description—Apothecia solitary, gregarious to caespitose, shallowly infundibuliform, with central to slightly eccentrical stipes, 3–13 mm in diam.; hymenium surface greyish green to blackish green when fresh, yellowish brown to greyish brown after drying; receptacle surface brown; stipe concolorous with the receptacle. Ectal excipulum of textura angularis, 20–55 μm thick; cells light brown to brown, nearly isodiametric or somewhat irregular, becoming smaller towards the surface, 3.5–15 μm in diam., some up to 22 × 14 μm, thin- to slightly thick-walled, giving rise to tomentum hyphae oriented nearly perpendicularly to the flank surface; tomentum hyphae clavate, 8–22 × 5–14 μm, walls up to 3.5 μm thick. Medullary excipulum of textura intricata, 30–860 μm thick; hyphae light brown to brown, 3–6 μm wide. Subhymenium not distinguishable. Hymenium 110–180 μm thick. Asci arising from croziers, 8-spored, cylindric-clavate, apex rounded, apical rings J+ in Melzer’s reagent and Lugol’s solution without KOH pretreatment, visible as two blue lines, 102–132 × 5.5–7.5 μm. Ascospores oblong ellipsoidal, somewhat inequilateral, with one side slightly flattened, non-septate, thin-walled, hyaline, smooth, with two large guttules and some small ones, filling most parts of the ascospores, irregularly biseriate, 8.5–14.5 × 3.5–4.5 μm. Paraphyses filiform, septate, simple, hyaline, 2.5–3.5 μm wide at apex and 2–3 μm wide below, equaling to or slightly exceeding the asci by 5–10 μm.

Paratypes—China, Xizang, Nyingchi, Lulang, on decorticated rotten wood, 23 September 2016, Huan-Di Zheng, Zhi-He Yu, Zhao-Qing Zeng et al. 11237 (HMAS 290935, ITS: PX453029, 28S: PX630172, RPB1: PX557844, RPB2: PX557851, TEF1: PX529000); ibid., 11238 (HMAS 275647, ITS: PX453030, 28S: PX630173, RPB1: PX557845, RPB2: PX557852, TEF1: PX529001); ibid., 11243 (HMAS 275645, ITS: PX453031, 28S: PX630174, RPB1: PX557846, RPB2: PX557853, TEF1: PX529002).

Additional specimens examined—China, Anhui, Luan, Shucheng, Wanfoshan National Forest Park, 31.0209° N, 116.3308° E, alt. 1316 m, on decorticated rotten wood, 17 October 2020, Xin Tao DBSAH2020101705 (HMAS 287036, as *C. torta*); Beijing, Donglingshan, alt. 1100 m, on decorticated rotten wood, 20 August 1998, Zheng Wang & Shuang-Lin Chen 0287 (HMAS 75866, as *C. torta*); ibid*.*, alt. 1150 m, on decorticated rotten wood, 19 August 1998, Zheng Wang & Xiao-Qing Zhang 0258 (HMAS 75865, as *C. torta*); Heilongjiang, Yichun, Dailing, Liangshui forest station, alt. 400–500 m, on decorticated rotten wood, 27 August 1996, Zheng Wang & Wen-Ying Zhuang 1282 (HMAS 71905, as *C. torta*); Hubei, Wufeng, Houhe, alt. 800 m, on decorticated rotten wood, 13 September 2004, Wen-Ying Zhuang & Chao-Yang Liu 5608 (HMAS 266515, as *C. torta*); Jilin, Dunhua, Huangnihe, Donggou, alt. 350 m, on decorticated rotten wood, 17 August 2000, Wen-Ying Zhuang & Zhi-He Yu 3559 (HMAS 78145, as *C. torta*); Yunnan, Xishuangbanna, Menghai, Mangao, on decorticated rotten wood, 20 October 1998, Wen-Ying Zhuang & Zhi-He Yu 3192 (HMAS 266513, as *C. torta*); Yunnan, Xishuangbanna, Mengla, on decorticated rotten wood, 18 October 1998, Wen-Ying Zhuang & Zhi-He Yu 3136 (HMAS 266514, as *C. torta*).

Known distribution—China (Anhui, Beijing, Heilongjiang, Hubei, Jilin, Xizang, Yunnan), South Korea.

Notes—*Chlorencoelia biguttulata* is morphologically similar to *C. torta* in features of excipular structure, asci, and ascospores, but they are phylogenetically distinct. Meanwhile, the two species can be differentiated by hymenium surface colour, which is olive yellow to olive green in *C. torta* and greyish green to blackish green in *C. biguttulata* [1]. This is the most commonly seen *Chlorencoelia* species in China. Specimens of *C. biguttulata* from China were misidentified as either *C. torta* or *C. versiformis* [15], but they were proven to be divergent from the authoritative materials of the two species in ITS sequence (Figure 1). Therefore, *C. torta* or *C. versiformis* ought to be removed from the Chinese species checklist. Three ITS sequences (JN033400, KR673605, and KR673699) derived from the South Korean materials were also wrongly identified as *C. torta*, for which *C. biguttulata* is the correct name.

#### 3.2.2. *Chlorencoelia ellipsoidea* H.D. Zheng & W.Y. Zhuang, sp. nov. (Figure 2d–f and Figure 4)

Fungal Names—FN 573050.

Etymology—The specific epithet refers to the ellipsoid ascospores of the fungus.

**Figure 4:**
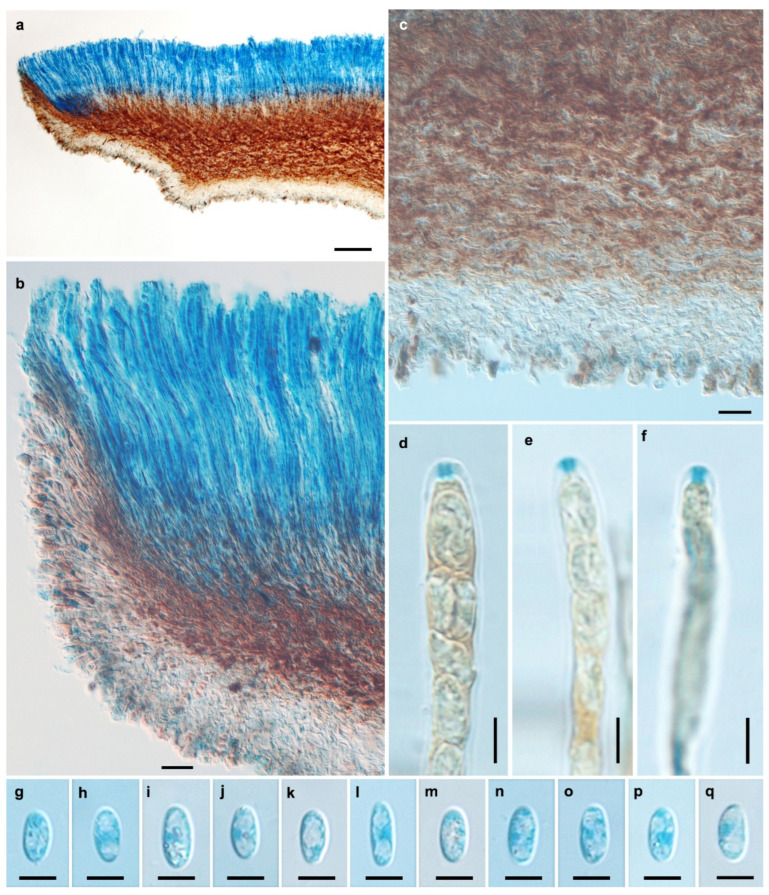
Microscopic features of *Chlorencoelia ellipsoidea* (HMAS 290936, holotype). (**a**) Longitudinal section of apothecium (partial); (**b**) hymenium and excipular structure of margin and flank; (**c**) excipular structure of flank; (**d**–**f**) IKI reaction of ascus apical ring without KOH pretreatment; (**g**–**q**) ascospores. Scale bars: (**a**) = 100 μm; (**b**,**c**) = 20 μm; (**d**–**q**) = 5 μm. Mounting media: (**a**–**c**,**g**–**q**) Lactophenol cotton blue; (**d**–**f**) Melzer’s reagent.

Holotype—China, Yunnan, Gaoligong Mountains, Baihualing, Jinchanghe, on decorticated rotten wood, 17 September 2017, Huan-Di Zheng, Xin-Cun Wang, Yu-Bo Zhang & Yi Zhang 11442 (HMAS 290936, ITS: PX453032, 28S: PX630175, RPB1: PX557847, RPB2: PX557854, TEF1: PX529003).

Chinese Name—椭孢绿散孢盘菌, tuǒ bāo lǜ sǎn bāo pán jūn.

Description—Apothecia solitary, gregarious to caespitose, shallowly infundibuliform, distinctly stipitate, 7–12 mm in diam.; hymenium surface atrovirens, dark green to blue green when fresh; receptacle surface brown, somewhat furfuraceous; stipe concolorous with the receptacle. Ectal excipulum of textura angularis, 40–105 μm thick; cells hyaline to light brown, 19–30 × 9–16.5 μm, some nearly isodiametric and 11–16.5 μm in diam., thin- to slightly thick-walled, giving rise to tomentum hyphae oriented nearly perpendicularly to the flank surface; tomentum hyphae clavate, 11–22 × 7–12 μm, walls 2–4 μm thick. Medullary excipulum of textura intricata, tightly arranged, 50–260 μm thick; hyphae brown, 2.5–5 μm wide. Subhymenium not distinguishable. Hymenium 205–220 μm thick. Asci arising from simple septa, 8-spored, cylindric-clavate, apex rounded, apical rings J+ in Melzer’s reagent and Lugol’s solution without KOH pretreatment, visible as two blue lines, 115–126 × 5.5–6 μm. Ascospores broad ellipsoidal, non-septate, thin-walled, hyaline, smooth, with 1–2 large guttules and some small ones, filling most part of the ascospores, uniseriate, 7.2–9.1 × 3.5–4.2 μm. Paraphyses filiform, septate, simple, hyaline, 3–3.5 μm wide at apex and 2–2.5 μm wide below, equaling to or slightly exceeding the asci by 5–10 μm.

Known distribution—China (Yunnan).

Notes—*Chlorencoelia ellipsoidea* is distinct from other members of the genus by dark green hymenium surface, distinct stipe and broad ellipsoidal ascospores. Phylogenetically, it is a sister species of *C. torta*, which is different in smaller apothecia, olive-yellow to olive-green hymenium surface, larger, biguttulate, and subcylindric-ellipsoidal ascospores [1].

#### 3.2.3. *Chlorencoelia sinensis* H.D. Zheng & W.Y. Zhuang, sp. nov. (Figure 2g–k and Figure 5)

Fungal Names—FN 573051.

Etymology—The specific epithet refers to the known geographic range of the fungus.

Holotype—China, Chongqing, Jinfo Mountain, on decorticated rotten wood, 29.0275° N, 107.1869° E, alt. 2130 m, 26 October 2020, Huan-Di Zheng, Zhao-Qing Zeng, Xin-Cun Wang & Chang Liu 12699 (HMAS 290937, ITS: PX453033, 28S: PX630176, RPB1: PX557848, RPB2: PX557855, TEF1: PX529004).

Chinese Name—中国绿散孢盘菌, zhōng guó lǜ sǎn bāo pán jūn.

Description—Apothecia solitary, gregarious to caespitose, occasionally 2–3 forming on a common base, discoid to shallowly cupulate, short-stipitate to sub-stipitate, 2–7 mm in diam.; hymenium surface yellowish green when fresh, greyish green after drying; receptacle surface nearly black; stipe concolorous with the receptacle. Ectal excipulum of textura angularis, 30–70 μm thick; oriented nearly perpendicularly to the receptacle surface, cells 5.5–11 μm in diam., walls brown, giving rise to filamentous (margin to upper portion) to clavate (flank to lower portion) tomentum hyphae, 6–19 × 5–11 μm, thick-walled, walls 1.5–5 μm thick. Medullary excipulum of textura intricata, 80–680 μm thick; hyphae brownish, 3–4.5 μm wide. Subhymenium not distinguishable. Hymenium 95–110 μm thick. Asci arising from croziers, 8-spored, cylindric-clavate, apex rounded, narrowed gradually into a short stalk, apical rings J+ in Melzer’s reagent and Lugol’s solution without KOH pretreatment, visible as two blue lines, 75–100 × 5.2–6 μm. Ascospores fusoidal, anterior end round, posterior end pointed, slightly flattened on one side, aseptate, thin-walled, hyaline, smooth, with two large guttules and some small ones, filling most part of the ascospores, irregularly biseriate, 9–13.7 × 2.2–3.5 μm. Paraphyses filiform, septate, simple, hyaline, 2.5–3.5 μm wide at apex and 2–3 μm wide below, equaling to or slightly exceeding the asci.

Paratype—China, Yunnan, Shangri La, Jiantang County, Potatso National Park, 27.8275° N, 99.9579° E, alt. 3670 m, on decorticated rotten wood, 30 September 2020, Xiang-Hua Wang 9258 (HMAS 290938, ITS: PX453034, 28S: PX630177, RPB1: PX557849).

Additional specimen examined—China, Anhui, Anqing, Yuexi, Miaodaoshan National Forest Park, 30.4953° N, 116.0528° E, alt. 622 m, on decorticated rotten wood, 19 October 2021, Lei Yao DBSAH20211019100 (HMAS 285422, as *C. torta*).

Known distribution—China (Anhui, Chongqing, Taiwan, Yunnan).

**Figure 5:**
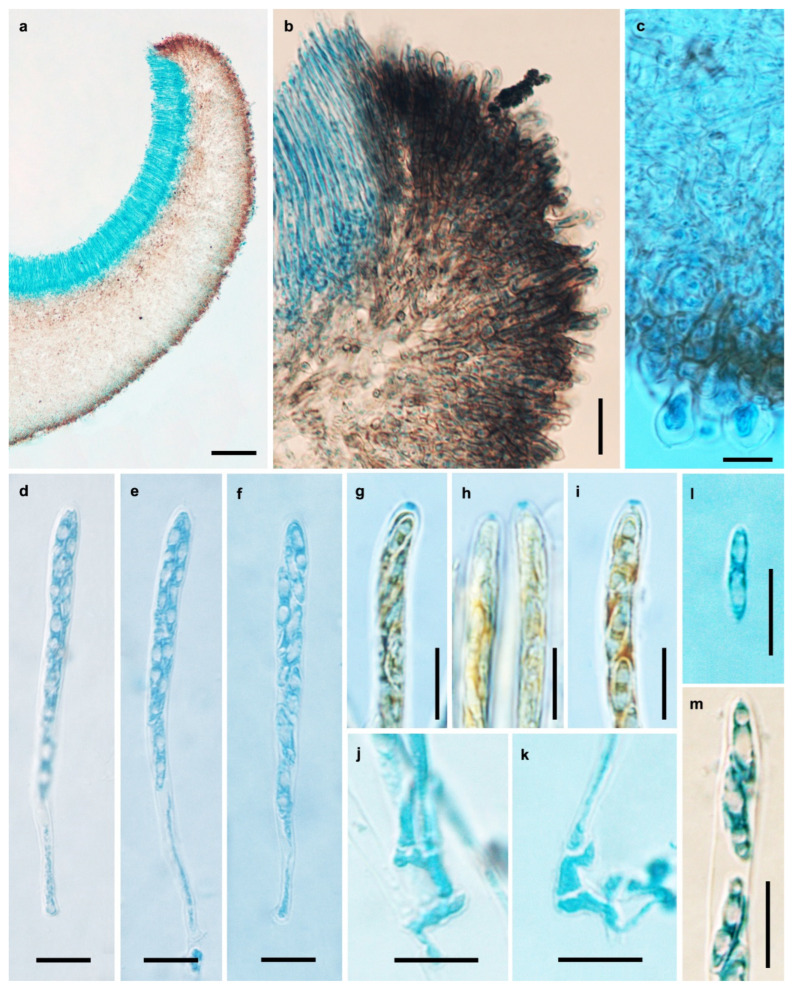
Microscopic features of *Chlorencoelia sinensis* (HMAS 290937, holotype). (**a**) Longitudinal section of apothecium (partial); (**b**) excipular structure of margin and flank; (**c**) excipular structure of flank; (**d**–**f**) asci; (**g**–**i**) IKI reaction of ascus apical ring without KOH pretreatment; (**j**,**k**) croziers at ascus bases; (**l**) ascospore; (**m**) ascospores in ascus. Scale bars: (**a**) = 100 μm; (**b**) = 20 μm; (**c**–**m**) = 10 μm. Mounting media: (**a**–**f**,**j**–**m**) Lactophenol cotton blue; (**g**–**i**). Melzer’s reagent.

Notes—*Chlorencoelia sinensis* is similar to *C. torta* in excipular structure and resembles *C. versiformis* in ascospore morphology [1], but it is distinct in the combination of substipitate apothecia, blackish receptacle surface and fusoidal ascospores. Collection of the species from Anhui Province (HMAS 285422) and ITS sequences generated from materials from Taiwan Province (LC862140, LC862114) were previously misidentified as *C. torta*. Phylogenetically, *C. sinensis* exhibits distant relationships with *C. torta* and *C. versiformis*, while it is relatively close to *C. olivacea*, *C. northlandica* and the three undescribed species (Figure 1)*. C. olivacea* is different in dark green or bluish hymenium surface and pale brown excipular cells. *C. northlandica* can be distinguished by dark green to grey-blue hymenium surface and broader ascospores (9.5–13 × 3–4.5 μm).

#### 3.2.4. Comparison of Morphological Characteristics Among *Chlorencoelia* Species

To give a straightforward comparison and facilitate recognition of *Chlorencoelia* species, the key morphological taxonomic characteristics are summarized in Table 2.

#### 3.2.5. Key to the Known Species of *Chlorencoelia*

1. Ascospores 20–40 × 4.5–6 μm, cylindric-fusoid
*C. macrospora*
1. Ascospores less than 20 μm long22. Ascospores pyriform, 5–8 × 1.5–3 μm
*C. ripakorfii*
2. Ascospores of other shapes, mostly longer than 8 μm33. Tomentum hyphae filamentous
*C. versiformis*
3. Tomentum hyphae clavate44. Ascospores eguttulate, ellipsoidal, 0–1 septate, 7.5–9.7 × 2.2–3.7 μm
*C. indica*
4. Ascospores guttulate55. Ascospores fusoidal, 9–13.7 × 2.2–3.5 μm
*C. sinensis*
5. Ascospores ellipsoidal66. Ascospores broad-ellipsoidal, length/width less than 2.2, 7.2–9.1 × 3.5–4.2 μm, medullary excipulum brown
*C. ellipsoidea*
6. Ascospores ellipsoidal, length/width more than 2.277. Ascospores ellipsoidal, 8.5–12 × 2.5–3.5 μm
*C. olivacea*
7. Ascospores oblong-ellipsoidal88. Tomentum hyphae barely differentiated, ascospores 9.5–13 × 3–4.5 μm
*C. northlandica*
8. Tomentum hyphae well-differentiated 99. Tomentum hyphae slightly thick-walled, ascospores relatively large, (9.5–)11–16.5 × (2.5–)3.5–5 μm
*C. australis*
9. Tomentum hyphae distinctly thick-walled, ascospores relatively small1010. Hymenium surface olive yellow to olive green when fresh, ascospores (5.6–)9–11(–12) × 2–4 μm
*C. torta*
10. Hymenium surface greyish green to blackish green when fresh, ascospores 8.5–14.5 × 3.5–4.5 μm
*C. biguttulata*


## 4. Discussion

Upon the establishment of *Chlorencoelia*, taxonomy of the genus, for a long time, was mainly based on morphological features of tomentum hyphae, ascus, and ascospores. Owing to the small apothecia and inconspicuous coloration, members of *Chlorencoelia* are not easily determined in the field; thus limited attention was paid to the genus and taxonomy of the genus was not well documented. Moreover, due to the morphological features of different species being very similar or overlapping, it is difficult to draw clear boundaries among species, which results in the existence of cryptic species. According to morphological traits, a considerable number of specimens from different parts of the world were identified as *C. torta* or *C. versiformis*, the two commonly known species. Nowadays, integrative analysis of molecular and morphological data facilitates accurate species identification. For example, *C. australis* and *C. northlandica* were separated from *C. torta* or *C. versiformis* based on DNA sequencing and morphological analyses of specimens from New Zealand [5]. Similar results can be found in the present study; *C. biguttulata* and *C. sinensis* were distinguished from *C. torta* or *C. versiformis* according to the combined data. As the number of species found increases, it becomes difficult to distinguish *Chlorencoelia* species merely by morphology, especially the species having clavate tomentum hyphae and ellipsoidal ascospores (*C. australis*, *C. biguttulata*, *C. northlandica*, *C. olivacea* and *C. torta*). Molecular inference plays an important role in accurate species recognition in addition to morphological observations.

As for DNA barcodes for species identification, only ITS sequences are available for most *Chlorencoelia* species, and the fragment has been proven to be efficient. It is unfortunate that ITS sequences of *C. indica*, *C. macrospora*, and *C. ripakorfii* are not available, and we cannot locate their phylogenetic positions (Figure 1). We have acquired the 28S, RPB1, RPB2, and TEF1 sequences of the new species; however, these DNA markers are not available for other species. Multi-gene phylogenetic analysis surely awaits the accumulation of more DNA fragments of the group.

In subsequent research, mycologists worldwide need to pay more attention to *Chlorencoelia*, carry out field surveys across a broader geographical range, cover diverse vegetation types, collect specimens from various substrates, isolate pure cultures, check morphological features in detail, conduct multi-locus phylogenetic analyses, and undertake systematic comparative studies. In doing so, an increasing number of novel species will be uncovered, the ecology and substrate preference of the genus will be better understood, and a comprehensive infrageneric framework of *Chlorencoelia* will be fully resolved in the future.

## Figures and Tables

**Table 1:** List of taxa with information on origin, voucher/strain, substrate, and GenBank accession numbers of ITS sequences used in phylogenetic analyses (numbers in bold indicating newly generated sequences).

Species	Origin	Voucher/Strain	Substrate	Accession Numbers
*Chlorencoelia australis*	New Zealand	ICMP 21732 ex type	Decorticated wood of *Leptospermum scoparium*	MH682234
*C. australis*	New Zealand	ICMP 25691	Rotting wood	PQ541258
*C. australis*	New Zealand	ICMP 25618	Dead wood	PQ533031
*C. australis*	Australia	ICMP 25304	Decorticated wood	PP701695
*C. biguttulata*	China, Xizang	HMAS 275646 holotype	Decorticated rotten wood	**PX453028**
*C. biguttulata*	China, Xizang	HMAS 275647 paratype	Decorticated rotten wood	**PX453030**
*C. biguttulata*	China, Xizang	HMAS 275645 paratype	Decorticated rotten wood	**PX453031**
*C. biguttulata*	China, Xizang	HMAS 290935 paratype	Decorticated rotten wood	**PX453029**
*C. biguttulata* (*C. “torta”*)	China, Anhui	HMAS 287036	Decorticated rotten wood	OQ534202
*C. biguttulata* (*C. “torta”*)	South Korea	KUS-F52256	Wood	JN033400
*C. biguttulata* (*C. “torta”*)	South Korea	KA13-1232	Unknown	KR673699
*C. biguttulata* (*Chlorencoelia* sp.)	South Korea	KA12-1413	Unknown	KR673605
*C. ellipsoidea*	China, Yunnan	HMAS 290936 holotype	Decorticated rotten wood	**PX453032**
*C. northlandica*	New Zealand	ICMP 21462 ex type	Decorticated wood	PQ541254
*C. northlandica*	New Zealand	ICMP 25719	Decorticated wood	PQ541259
*C. olivacea*	New Zealand	ICMP 25720 ex type	Decaying wood of *Fuscospora fusca*	PQ541260
*C. olivacea*	New Zealand	PDD 106117	Wood of *Fuscospora fusca*	OR565293
*C. olivacea*	New Zealand	ICMP 25721	Decorticated wood *Fuscospora fusca*	PQ541256
*C. olivacea*	New Zealand	PDD 106302	Rotting stump of *Fuscospora fusca*	MK432802
*C. olivacea*	New Zealand	PDD 106239	Rotting wood of *Fuscospora fusca*	MK432798
*C. olivacea*	New Zealand	PDD 122841	Rotting wood	PQ533030
*C. olivacea*	New Zealand	ICMP 24559	Decorticated wood	PQ541257
*C. olivacea*	New Zealand	ICMP 24001	Decorticated wood of Nothofagaceae sp.	PQ541255
*C. olivacea*	New Zealand	PDD 114302	Wood of *Fuscospora cliffortioides*	OR565298
*C. sinensis*	China, Chongqing	HMAS 290937 holotype	Decorticated rotten wood	**PX453033**
*C. sinensis*	China, Yunnan	HMAS 290938 paratype	Decorticated rotten wood	**PX453034**
*C. sinensis* (*C. “torta”*)	China, Anhui	HMAS 285422	Decorticated rotten wood	OQ534201
*C. sinensis* (*C. “torta”*)	China, Taiwan	SG2212_07_M1	Rotten wood	LC862114
*C. sinensis* (*C. “torta”*)	China, Taiwan	SG2311_04	Rotten wood	LC862140
*C. torta*	USA	S.D. Russell iNaturalist # 91595985	Unknown	OM809323
*C. torta*	USA	S.D. Russell iNaturalist # 91584233	Unknown	OM809329
*C. torta*	USA	S.D. Russell iNaturalist # 92192624	Unknown	OM809252
*C. torta*	USA	TU:119690	Rotten wood	LT158481
*C. torta*	USA	TU:119681	Rotten wood	LT158480
*C. torta*	USA	ZW-Geo55-Clark	Unknown	AY789352
*C. versiformis*	Estonia	TU:104550	Rotten wood	LT158411
*C. versiformis*	Estonia	TAAM:179803	Rotten wood	LT158427
*C. versiformis*	UK	HFRG_JN241028_1	Unknown	PV690292
*C. versiformis*	Canada	TRTC 175704	Unknown	PP386638
*C. versiformis*	USA	S.D. Russell iNaturalist # 8552320	Unknown	OM747569
*C. versiformis*	USA	S.D. Russell iNaturalist # 8552320	Unknown	MN906214
*C. versiformis*	Canada	DAOMC 251598	Decaying hardwood log	MH457140
*C. versiformis*	USA	TU:119720	Rotten wood	LT158479
*C. versiformis*	Canada	BIOUG24046-D05	Unknown	KT695372
*Chlorencoelia* sp.1 (*C. “torta”*)	Thailand	KMP3-7	Unknown	OP644990
*Chlorencoelia* sp.2 (cf. *Chlorencoelia* sp.)	Chile	MES-2558	Wet wood	MH930348
*Chlorencoelia* sp.3 (*C. “torta”*)	China, Taiwan	KL167_HB8415	Rotten deciduous wood	LT158424
*Hymenoscyphus fraxineus*	China, Jilin	HMAS 264174	Petioles and leaf veins of *Frexinus mandschurica*	KF188724
*H. fructigenus*	China, Hubei	HMAS 275508	Rotten fruit	KY498615

**Table 2:** Comparisons of morphological characteristics of *Chlorencoelia* species.

Species	Colour of Hymenium/Receptacle	Apothecium Size	Stipe	Tomentum Hyphae	Ascus Size	Ascospore		
						Shape	Size	Guttulation
*C. australis* [5]	Steel blue or green	5–10 mm	Short and broad stipitate	Clavate	90–135 × 9–12 μm	Oblong-elliptic	(9.5–)11–16.5 × (2.5–)3.5–5 μm	2–3 large guttules
*C. biguttulata*	Greyish green to blackish green	3–13 mm	Stipitate, central to somewhat eccentrical	Clavate	102–132 × 5.5–7.5 μm	Oblong ellipsoidal, somewhat inequilateral	8.5–14.5 × 3.5–4.5 μm	2 large guttules
*C. ellipsoidea*	Atrovirens, dark green to blue green	7–12 mm	Stipitate	Clavate	115–126 × 5.5–6 μm	Ellipsoidal	7.2–9.1 × 3.5–4.2 μm	1–2 large guttules
*C. indica* [2,30]	Dark greyish brown	3–15 mm	Lateral and short stipitate	Club-shaped	92–123 × 4.5–6.3 μm	Ellipsoid	7.5–9.7 × 2.2–3.7 μm	2 large guttules
*C. macrospora* [4]	Greenish grey to greenish black	5–8 mm	Stipitate	Filamentous, straight or coiled	160–230 × 9–13 μm	Cylindric-fusoid	20–40 × 4.5–6 μm	Many guttules
*C. northlandica* [5]	Dark green to grey-blue	5–10 mm	Short and broad stipitate	Globose to subclavate	90–127 × 5.5–7 μm	Oblong-elliptic	9.5–13 × 3–4.5 μm	2 large guttules
*C. olivacea* [5]	Olive green to dark blue-green	Up to 10.5 mm	Short and broad stipitate	Globose to subclavate	75–104 × 5–9 μm	Elliptic	8.5–12 × 2.5–3.5 μm	2–3 large guttules
*C. ripakorfii* [3]	Whitish yellow	2–5 mm	Stipitate	Cylindrical to slightly subclavate	(42–)45–64 × 3–4 μm	Ellipsoid, sigmoid or pyriform	5–8 × 1.5–3 μm	1–2 small guttules
*C. sinensis*	Yellowish green	2–7 mm	short-stipitate to sub-stipitate	Filamentous at margin, clavate at flank	75–100 × 5.2–6 μm	Fusoidal	9–13.7 × 2.2–3.5 μm	2 large guttules
*C. torta* [1,5]	Olive yellow to olive green	3–7 mm	stipitate	Subglobose to clavate	(69–)90–121(–126) × 5–7 μm	Irregularly ellipsoid	(5.6–)9–11(–12) × 2–4 μm	2 large guttules
*C. versiformis* [1]	Olive yellow to olive green	7–9 mm	stipitate	Filamentous to slightly clavate	(79–)95–130 (–150) × 5–8 μm	Cylindric-oblong	(10–)11–15 × 2.5–3.5 μm	Up to 4 guttules

## Data Availability

The newly generated sequences were deposited in GenBank; accession numbers are included in the article (https://www.ncbi.nlm.nih.gov/genbank, accessed on 9 October 2025 and 11 November 2025; Supplementary Files S1–S5). Names of the new species were formally registered in the global data repository Fungal Names (https://nmdc.cn/fungalnames, accessed on 10 October 2025). The original contributions presented in this study are included in the article. Further inquiries can be directed to the corresponding author.

## Supplementary Materials

The following supporting information can be downloaded at: https://www.mdpi.com/article/10.3390/jof11120855/s1, Supplementary File S1: Newly generated ITS sequences; Supplementary File S2: Newly generated 28S sequences; Supplementary File S3: Newly generated RPB1 sequences; Supplementary File S4: Newly generated RPB2 sequences; Supplementary File S5: Newly generated TEF1 sequences.
